# Isolation, characterization, and fibroblast uptake of bacterial extracellular vesicles from *Porphyromonas gingivalis* strains

**DOI:** 10.1002/mbo3.1388

**Published:** 2023-10-16

**Authors:** Helene R. Haugsten, Anne K. Kristoffersen, Trude M. Haug, Tine M. Søland, Reidun Øvstebø, Hans C. D. Aass, Morten Enersen, Hilde K. Galtung

**Affiliations:** ^1^ Institute of Oral Biology, Faculty of Dentistry University of Oslo Oslo Norway; ^2^ Department of Pathology Oslo University Hospital Oslo Norway; ^3^ The Blood Cell Research Group, Department of Medical Biochemistry Oslo University Hospital Ullevål Norway

**Keywords:** bacterial vesicle, *Porphyromonas gingivalis*, virulence factors

## Abstract

Periodontitis is an inflammatory condition caused by bacteria and represents a serious health problem worldwide as the inflammation damages the supporting tissues of the teeth and may predispose to systemic diseases. *Porphyromonas gingivalis* is considered a keystone periodontal pathogen that releases bacterial extracellular vesicles (bEVs) containing virulence factors, such as gingipains, that may contribute to the pathogenesis of periodontitis. This study aimed to isolate and characterize bEVs from three strains of *P. gingivalis*, investigate putative bEV uptake into human oral fibroblasts, and determine the gingipain activity of the bEVs. bEVs from three bacterial strains, ATCC 33277, A7A1‐28, and W83, were isolated through ultrafiltration and size‐exclusion chromatography. Vesicle size distribution was measured by nano‐tracking analysis (NTA). Transmission electron microscopy was used for bEV visualization. Flow cytometry was used to detect bEVs and gingipain activity was measured with an enzyme assay using a substrate specific for arg‐gingipain. The uptake of bEVs into oral fibroblasts was visualized using confocal microscopy. NTA showed bEV concentrations from 10^8^ to 10^11^ particles/mL and bEV diameters from 42 to 356 nm. TEM pictures demonstrated vesicle‐like structures. bEV‐gingipains were detected both by flow cytometry and enzyme assay. Fibroblasts incubated with bEVs labeled with fluorescent dye displayed intracellular localization consistent with bEV internalization. In conclusion, bEVs from *P. gingivalis* were successfully isolated and characterized, and their uptake into human oral fibroblasts was documented. The bEVs displayed active gingipains demonstrating their origin from *P. gingivalis* and the potential role of bEVs in periodontitis.

## Graphical abstract

Bacterial extracellular vesicles (bEVs) from *Porphyromonas gingivalis* were isolated and characterized before uptake into oral fibroblast was visualized. bEVs from *P. gingivalis* displayed active gingipains that may have a role in the pathogenesis of periodontitis.

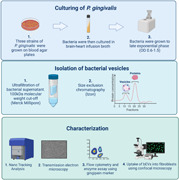

## 1 INTRODUCTION

Extracellular vesicles (EVs) are nanoparticles released by cells, including bacteria and protozoans (Gill et al., 2019). EVs carry biomolecules such as nucleic acids, proteins, and lipids (Yáñez‐Mó et al., 2015). Over the last decade, the research field of EVs has expanded tremendously.

Once considered cellular debris, EVs are now recognized as important in both physiological and pathological processes, including cell‐to‐cell communication, transfer of molecular cargo from one cell to another, and involvement in immune responses (Yáñez‐Mó et al., 2015). The vesicle cargo is heterogeneous, and the function of the vesicles is content‐dependent and varies with the cell of origin.

Pathogenic and nonpathogenic, gram‐positive, and gram‐negative bacteria all produce and secrete bacterial extracellular vesicles (bEVs) constitutively (Jan, 2017). The bacteria produce bEVs by outward budding of the bacterial membrane, although their biogenesis in gram‐positive bacteria is yet incompletely understood (Avila‐Calderón et al., 2021). In this manner, bacterial content such as lipids, nucleic acids, proteins, and virulence factors are kept inside and on the outer membrane of the vesicle during biogenesis, and this ensures a protected transport of the cargo out of the cell and into the extracellular surroundings. Thus, bEVs can function as a vehicle for bioactive molecules and may trigger host inflammation when invading host cells (Ho et al., 2015).

Periodontitis is a chronic inflammation that influences the supporting tissues of the teeth and may lead to partial or total tooth loss in the affected individuals if not treated (Socransky et al., 1998). Of importance, bacteria involved in periodontitis are also linked to the pathogenesis of several systemic conditions such as cardiovascular diseases, Alzheimer's disease, and diabetes (Bui et al., 2019; Zhang et al., 2021). Periodontitis is common worldwide, and approximately 50% of adults in the Western world have some degree of this ailment (Global, regional, and national incidence, prevalence, and years lived with disability for 328 diseases and injuries for 195 countries, 1990–2016: a systematic analysis for the GBD 2016 Disease and Injury Incidence and Prevalence Collaborators (2017)). It is a polymicrobial disease elicited by a complex of bacterial species that interacts with the host tissues and contributes to the destruction of tooth‐supporting tissues (Lamont et al., 2018). *Porphyromonas gingivalis*, an anaerobic bacterial species frequently isolated from patients with periodontitis, is an opportunistic bacterium and part of the commensal oral microbiota (Amano, 2003; Holt & Ebersole, 2005). It produces a range of virulence factors, many of them well‐known. Among them are gingipains, a cysteine protease group of the caspase family, the major (FimA) and minor fimbriae (Mfa1), lipopolysaccharides (LPS), capsule, and others (Lunar Silva & Cascales, 2021; Mysak et al., 2014). There is a large degree of genetic diversity among *P. gingivalis* strains and a high recombination in specific virulence genes such as fimbria genes, making some strains more virulent than others (Tribble et al., 2013). The gingipains, the virulence factors particularly important for this study, consist of arg‐gingipain (RgpA and RgpB), analyzed in this study, and lys‐gingipain (Kgp), both important for the pathogenesis of periodontitis (O'Brien‐Simpson et al., 2003; Sheets, 2008). Furthermore, the fimbriae play an important role in adherence and interaction with other bacteria as it is important for the mediation of the immune‐modulatory capacity of the bacteria (Brunner et al., 2010).

*P. gingivalis* has been shown to secrete a large number of bEVs considered to be a contributor to bacterial survival in the oral cavity and to the pathogenesis of periodontitis (Ho et al., 2015). It has been found that bacterial proteins, lipids, and DNA/RNA are present in the bEVs, including fragments encoding for gingipain and other virulence factors (Haurat et al., 2011; Veith et al., 2014). Virulence factors in bEVs have been described and found in specific high‐density clusters as in bacterial membranes (Veith et al., 2014) and both DNA and RNA fragments of fimbriae and gingipains are identified in bEVs from *P. gingivalis* strains (Ho et al., 2015). Extracellular stimuli like signaling between bacteria in biofilms (Ho et al., 2015) and nutritional conditions such as hemin concentration (Veith et al., 2018) can regulate vesicle production. Mantri et al. discovered a higher concentration of Hgp44 (an adhesion domain of rgpA) in bEVs from *P. gingivalis* than in the membrane of the bacteria itself, suggesting a larger amount of gingipains present in the bEVs (Mantri et al., 2015). The vesicles have, like their origin bacteria, the ability to invade host cells (O'Donoghue & Krachler, 2016) such as oral fibroblasts (Ho et al., 2015) and epithelial cells (Furuta, Takeuchi, et al., 2009; Furuta, Tsuda, et al., 2009) and can release cargo such as virulence factors inside the cell and thus modulate the host cell immune response (Mantri et al., 2015). It is expected that vesicles from different strains may differ with regard to binding and uptake into host cells.

**Figure 1:**
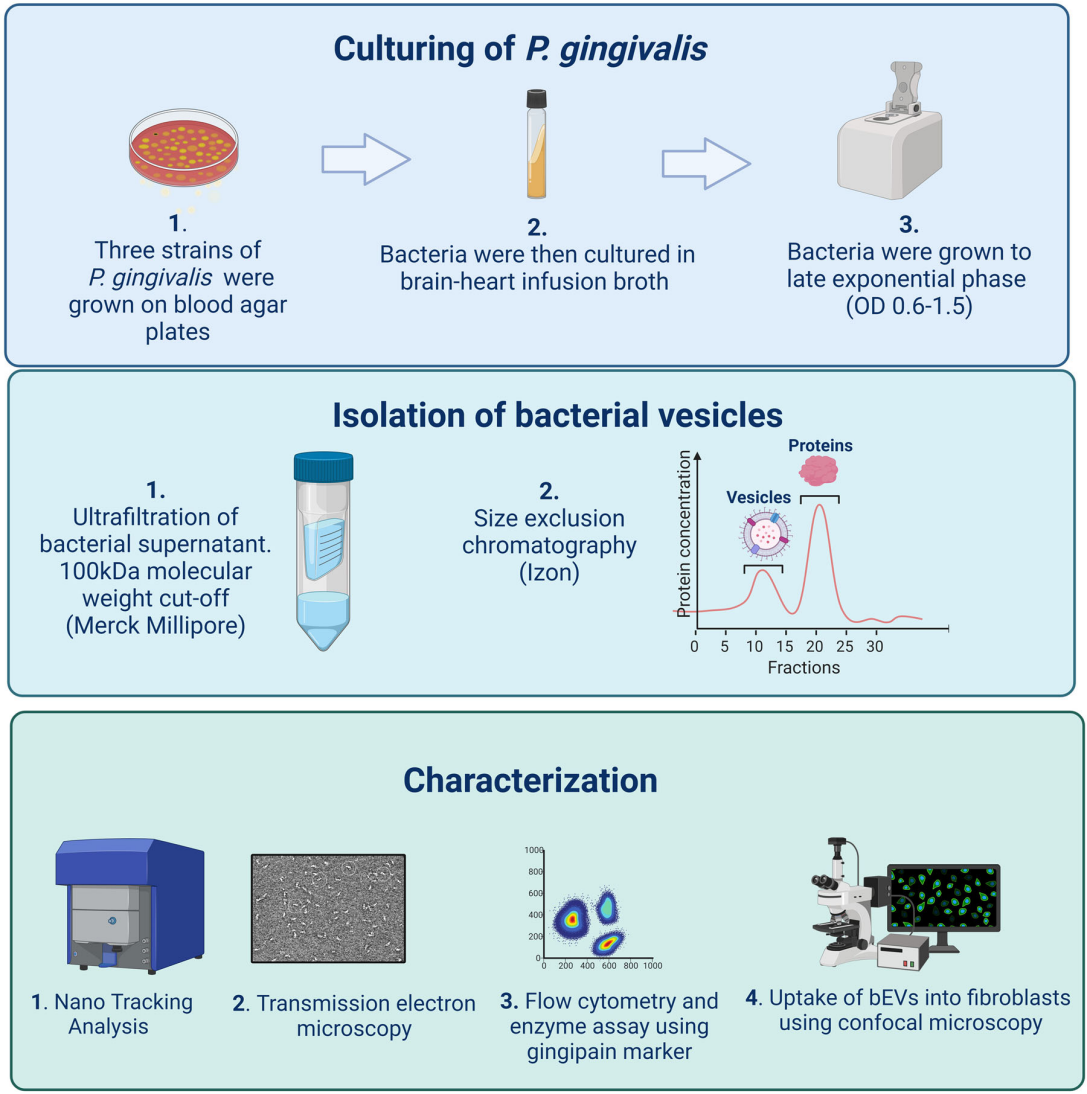
Overview of culturing of *Porphyromonas gingivalis*, isolation of bacterial extracellular vesicles (bEVs), and characterization of bEVs. Created with BioRender.

In this study, we aimed to isolate and characterize bEVs from three different strains of *P. gingivalis*; one clinical and two laboratory strains. We also wanted to study the uptake of *P. gingivalis* bEVs into human oral fibroblasts, as uptake of bEVs can induce cytokine secretion from fibroblasts and thus dysregulation of the inflammatory process in gingival tissue (Bartruff et al., 2005) and also induce various responses in different host cells. The presence and the activity of the virulence factor arg‐gingipain (rgpA and rgpB) in bEVs was also explored, to indicate if the vesicles were biologically active and specific for the bacteria. An overview of the study is illustrated in Figure 1. First, we isolated and characterized the bEVs, before investigating their uptake into oral fibroblasts, the most predominant cells in the supporting tissue of the teeth. The vesicles were characterized using nano‐tracking analysis (NTA), flow cytometry, and transmission electron microscopy (TEM). Furthermore, we studied the uptake of fluorescently labeled bEVs into normal oral fibroblasts by confocal imaging. Finally, we explored the presence and possible activity of the virulence factor arg‐gingipain in bEVs in the three strains, which further may help explain the roles of bEVs in the development of periodontal disease.

## 2 METHODS

### 2.1 Choice of strains and bacterial culturing

We chose *P. gingivalis* strains ATCC 33277, A7A1‐28, and W83 that represent low and high virulence capacity, based on variations in virulence regarding the large degree of genetic diversity of *P. gingivalis*. Examples of their characteristics are listed in Table 1. ATCC 33277, with *fimA I* genotype and no capsule, represents a strain with low virulence, but together with its high vesiculation capacity, it may be considered more virulent than earlier anticipated (Eick et al., 2006; Ho et al., 2016; Xie, 2015; Xu et al., 2018). W83, a *fimA* genotype IV, is encapsulated and gives a persistent immune response, thus representing a highly virulent strain. A7A1‐28, *fimA* genotype II, and with capsule also represents a high virulent strain, but with different capsules and fimbria (*FimA* and *Mfa1*) genotypes compared to W83. *Mfa1* is expressed in ATCC33277 and A7A1‐28 but with different genotypes. The capsule encloses the bacterial cell and is important for bacterial survival as it protects bacteria from phagocytosis and helps with bacterial evasion of host immune system activation, thus increasing virulence (Brunner et al., 2010; Singh et al., 2011). The fimbriae play an important role in adherence and interaction with other bacteria as it is important for the mediation of the immune‐modulatory capacity of the bacteria (Coats et al., 2019).

**Table 1:** Overview of strains of *Porphyromonas gingivalis* and virulence factors.

Virulence factor	ATCC 33277	A7A1‐28	W83
RgpB genotype	DYPN	NYPN	NSSK
FimA	I	II	IV
Mfa1 genotype	70A	53	Neg
Capsule	K‐	K3	K1
Virulence	Low virulence	High virulence	High virulence

The *P. gingivalis* strains ATCC 33277, A7A1‐28, and W83 were grown on blood agar plates under anaerobic conditions (37°C, 90% N_2_, 5% H_2_, 5% CO_2_). The three strains were selected from a collection at the Institute of Oral Biology, Faculty of Dentistry, University of Oslo, originally provided by A. J. van Winkelhoff, Academic Center for Dentistry Amsterdam (ACTA), Amsterdam, The Netherlands. These strains are described in detail in Enersen et al. (2008), Tribble et al. (2013), and Aduse‐Opoku et al. (2006). After 5 days of growth, pure, black‐pigmented colonies appeared on the blood agar plate.

At this point, stock solutions were made by placing the colonies in the Brain Heart Infusion broth (BHI, Thermo Fisher Scientific) supplemented with 100 µL 0.5 mg/mL Hemin and 100 µL 0.05 mg/mL vitamin K_3_ (Menadione). Then, for standardization, these stock solutions were added to the fresh BHI broth to a target OD of 0.08–0.1 was reached. This was controlled using Nanodrop (Absorbance 600 nm, Nanodrop 2000c, Thermo Fisher Scientific) at wavelength 600 nm. At this point, these bacteria in BHI broth were cultivated anaerobically until OD 0.6–1.5 (600 nm) was reached. This procedure was followed for all samples of all three strains. Samples with OD over 1.5 were excluded. As a negative control BHI broth supplemented with 100 µL 0.5 mg/mL Hemin and 100 µL 0.05 mg/mL vitamin K_3_ (Menadione) (no bacteria) was simultaneously kept in the incubation chamber. The supernatant was harvested after the removal of the bacteria by centrifugation at 3300 rpm for 30 min for all samples.

### 2.2 Isolation of vesicles

The bEVs were isolated as described by Guerreiro et al. (2018). Briefly, the supernatants were centrifuged at 1500*g* for 45 min at 20°C to remove large particles. The supernatants were then both concentrated and filtered by ultrafiltration (UF) using a 100 kDa molecular weight cut‐off Amicon‐Ultra15 Centrifugal Filter Unit (Merck Millipore) to a final volume of 500 µL, as recommended in the manufacturer's manual.

The vesicles from the three strains were then isolated from each aliquot of 500 µL volume with size exclusion chromatography (SEC) using qEV original columns (IZON), according to the manufacturer's instructions. SEC separates molecules based on size, where larger components like large vesicles are eluted first, and smaller components like proteins are eluted later. We eluted 30 fractions with sterile‐filtered phosphate‐buffered‐saline (PBS) from the SEC columns, and for each fraction, the protein concentrations were determined by spectrophotometry (Absorbance 280 nm, Nanodrop 2000c, Thermo Fisher Scientific). Here, we identified two separate peaks with different protein concentrations. Based on previous experience, the vesicles were expected to be in the fractions of the first peak, and proteins and other molecules were expected to be in the second peak, enabling us to collect the fractions with bEVs in the first peak (illustrated in Figure 1). As a negative control, BHI broth alone was processed the same way as the bacterial samples, with UF and SEC. The negative controls were the same for all three strains (from now only mentioned as negative control). Eluted fractions were stored at −80°C.

### 2.3 Characterization of vesicles

The concentration and size distribution of particles in collected fractions after SEC were measured using nano‐tracking analysis (NTA). The analyses were carried out on a NanoSight NS500 instrument (Malvern Instruments) with a 488 nm laser, a high‐sensitivity sCMOS camera, and a syringe pump. Samples were diluted with PBS to obtain a concentration within the range of 10^7^ and 10^9^ particles/mL. The camera level was set to 14, the detection threshold to 3, and three video replicates per sample were recorded. The analysis was performed with NTA software 3.4 Build 3.4.003 (Malvern).

TEM was used to verify the presence of vesicle‐like structures. The samples (15 µL) were gently placed on 100 mesh carbon‐coated copper grids (Electron Microscopy Science) for 5 min before being washed and incubated in 4% uranyl acetate for 5 min. The stained grids were left overnight to air dry at room temperature and then examined using a Philips CM120 BioTwin transmission electron microscope.

Flow cytometry was used to detect and characterize bEVs derived from *P. gingivalis* using antibodies against rgpB (anti‐rgpB, SC1195, Genescript, USA Inc). First, bEVs from all three strains (*n* = 3 per strain) and negative control (*n* = 2) were incubated with rgpB‐antibodies. Protein G magnetic beads (Dynabeads Protein G, Thermo Fisher Scientific) that bind to rgpB‐antibodies were then incubated with both bEVs and negative control. The samples were further stained with PKH67 (a fluorescent dye that stains membranes by intercalating their aliphatic portions into the exposed lipid bilayer) according to the manufacturer's protocol (Sigma Aldrich) to verify the binding of rgpB‐positive bEVs. The control samples were treated equal to and in parallel with the bacterial cultures, followed by an identical downstream methodology for retrieving and isolating the bEVs. Equal volumes (50 µL) of bEVs derived from samples and negative control were subjected to overnight incubation with beads. Magnetic beads were identified and gated in the SSC‐A versus FSC‐A dot plot. Single magnetic beads were captured in a pulse width versus SSC height dot plot, and the fluorescence of the PKH67 membrane staining was collected in the 530/30 detector. All samples were compared to each other in a histogram overlay of the area pulse of PKH67 fluorescence (FlowJo, Version 4‐10.9.0. Ashland, OR: Becton, Dickinson and Company; 2023). Samples were sonicated for 10 s to separate aggregated magnetic beads before flow cytometry analysis using a BD Accuri C6 (BD Bioscience). Delta median fluorescent intensity (ΔMFI) was calculated by subtracting the negative control beads from the magnetic bead captured rgpB positive bEVs.

### 2.4 Gingipain activity

Gingipain protease activity was determined using a peptide substrate, Gly‐Ala‐Arg‐d‐Arg‐Lys (synthesized by Biomatik Corporation), with N‐terminus fluorescence (TAMRA) and the quencher DABCYL linked to the c‐terminus, that reacts specifically with arg‐gingipains (Kaman et al., 2012). Proteolytic activity was determined by the addition of 1.5 µL gingipain substrate solution (0.8 mM) to 80 µL bEV or control samples in 96 well plates. We used BHI broth after UF and SEC as a negative control and fresh *P. gingivalis* strain 195PG3 (Enersen et al., 2006) as a positive control. Additionally, gingipain activity was determined in whole bacteria of ATCC 33277, A7A1‐28, and W83 grown on plates for 4 days and diluted in 0.045% NaCl to a comparable OD600, to confirm gingipain activity in those strains. Plates were read for 30 min with a 5‐min interval on a fluorescence reader (GEN 5.1, BioTek instruments Inc.) with an excitation wavelength of 485 nm and emission wavelength of 528 nm, according to the manufacturer's protocol. The protease activity was determined as relative fluorescence per minute. The blocking substrate carbobenzoxy‐Lys‐Arg‐CO‐Lys‐N‐(CH_3_)_2_ (KYT‐1) (synthesized by PeptaNova GmbH) (Kadowaki et al., 2004) which is an inhibitor of rgpA and rgpB was used to inhibit the activity of the protease and to validate the enzyme reaction.

### 2.5 bEV uptake into fibroblasts

Oral gingival fibroblasts were provided as a kind gift from Professor Daniela Costea, University of Bergen, Norway. The oral fibroblasts at passages 3–19 were cultured in 10% exosome‐depleted medium (Gibco, Life Technologies; Guerreiro et al., 2018) at 37°C in 5% CO_2_ until confluence, before they were trypsinated and centrifuged for 5 min at 1000*g*. A volume of 100 µL solution with ~5000 fibroblasts was seeded onto coverslips and allowed to attach and grow overnight. Vesicles from all three strains at a concentration of 10^8^–10^10^ particles/mL were stained with PKH67 (Sigma Aldrich) and incubated with fibroblasts on coverslips for 4 h in the exosome‐depleted medium. The cells were then fixed with PFA (Paraformaldehyde, Sigma Aldrich), and mounting media (SouthernBiotech) containing the nuclear stain DAPI was added before they were analyzed using a confocal microscope (Leica TCS SP8) at the Advanced Light Microscopy Core Facility at Oslo University Hospital (OUS).

### 2.6 Statistical analysis

Statistical analysis was performed using Graph Pad Prism 9.5.1 (733). The statistical significance of arg‐gingipain activity was calculated using Student's *t*‐test at a 5% significance level.

## 3 RESULTS

### 3.1 Vesicle concentration, size, and morphology

The panel of characterization techniques identified bEVs from all three strains of *P. gingivalis*. The protein content of each fraction was measured to be from 0.006 mg/mL up to 1.113 mg/mL for the bacterial strains and was not detectable for the negative control samples.

**Figure 2:**
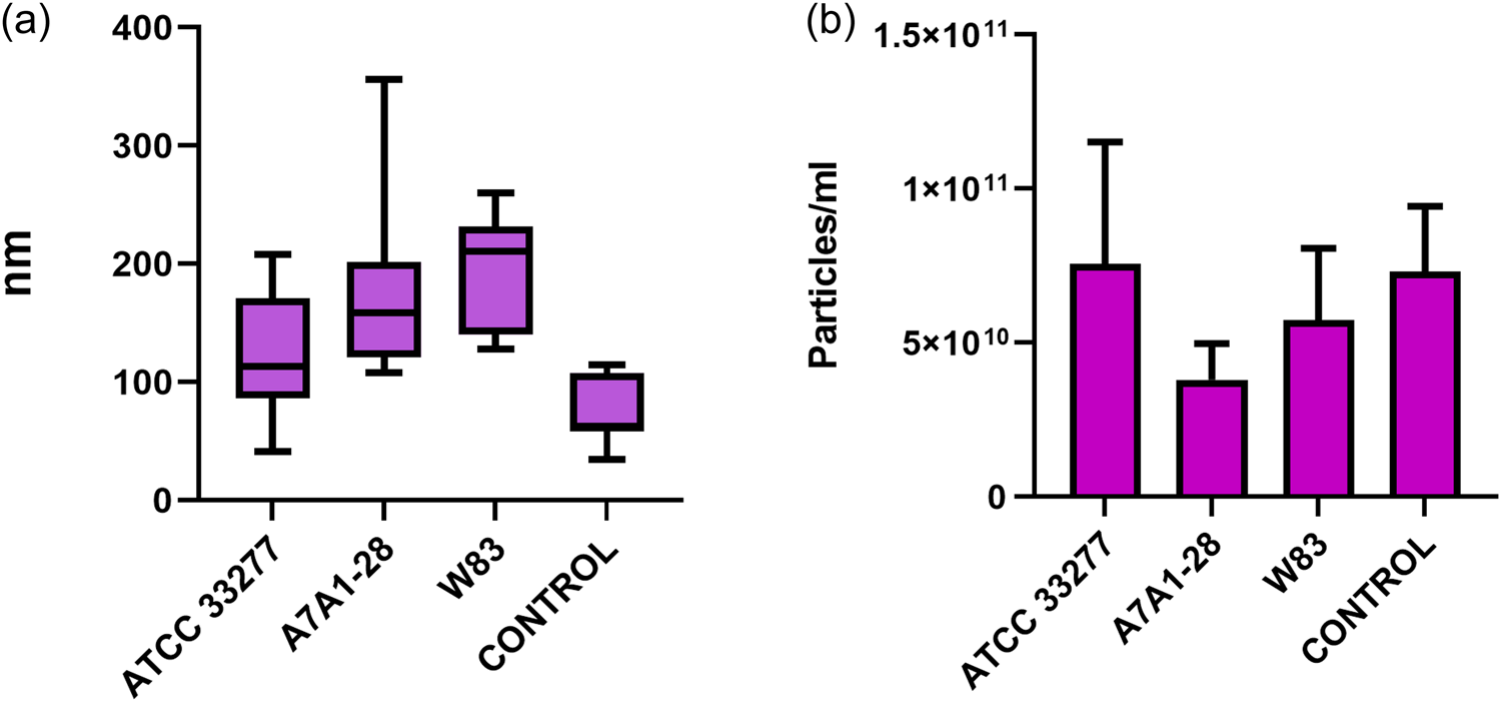
(a) Size of vesicle‐like particles from three strains of *Porphyromonas gingivalis* and negative control as measured with nano‐tracking analysis (NTA). The number of samples were between 18 and 13, for ATCC 33277 *n* = 13, A7A1‐28 *n* = 12, and W83 *n* = 13, respectively, and for negative control *n* = 8. (b) Concentration of vesicle‐like particles from three strains of *P. gingivalis* and negative control as measured with NTA.

NTA demonstrated particle concentrations of bEVs from 6.66 × 10^8^ particles/mL up to 1.51 × 10^11^ particles/mL, with a mean particle concentration of 5.67 × 10^10^ particles/mL (*n* = 38) (Figure 2). The negative control samples had particle concentrations from 2.31 × 10^9^ particles/mL to 1.66 × 10^11^ particles/mL, with a mean of 7.3 × 10^10^ particles/mL (*n* = 7) (Figure 2). The mean size of the vesicle‐like particles differed from 42 to 356 nm and varied both between samples of the same strain and between the three strains (Figure 2 and Table 2). The negative control samples showed a mean particle size of 76 nm.

**Table 2:** Overview of the size of bacterial extracellular vesicles (bEVs) from three strains of *Porphyromonas gingivalis* and negative control as measured with nano‐tracking analysis (NTA).

Particle size (nm)	ATCC 33277	A7A1‐28	W83	Negative control
n	13	12	13	7
Minimum	41.5	107.9	128.2	34.9
Maximum	207.8	355.8	260.0	114.8
Mean	121.1 ± 14.0	171.3 ± 20.0	196.6 ± 13.3	76.66 ± 11.3

**Figure 3:**
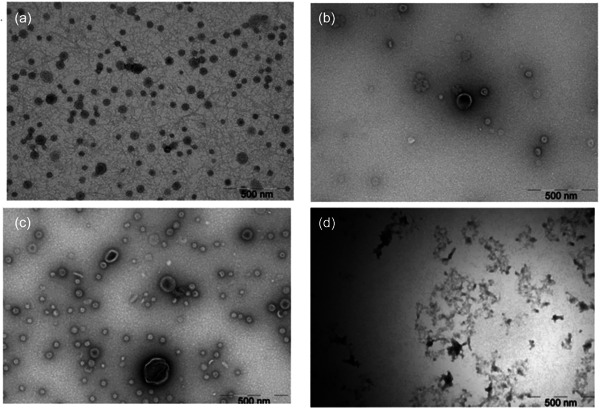
Representative transmission electron microscopy (TEM) pictures with vesicle‐like particles in samples with bacterial extracellular vesicles (bEVs) from (a) ATCC 33277, (b) A7A1‐28, (c) W83, and with no vesicle‐like particles in (d) the negative control. All the pictures are taken at magnification ×65,000.

Vesicle‐like particles with nano‐spherical shapes were visualized using TEM in the samples from the bacterial strains. The diameters varied from 20 to 120 nm (Figure 3) when evaluating images at magnification ×65,000. The vesicle morphology was heterogeneous, with various shapes and sizes. Vesicle aggregates were also identified. There were no differences in diameter and morphology that were consistent between the three strains when we explored the TEM pictures. Importantly, in the negative control samples, no vesicle‐like structures were present.

### 3.2 Verification of gingipains in bEVs

**Figure 4:**
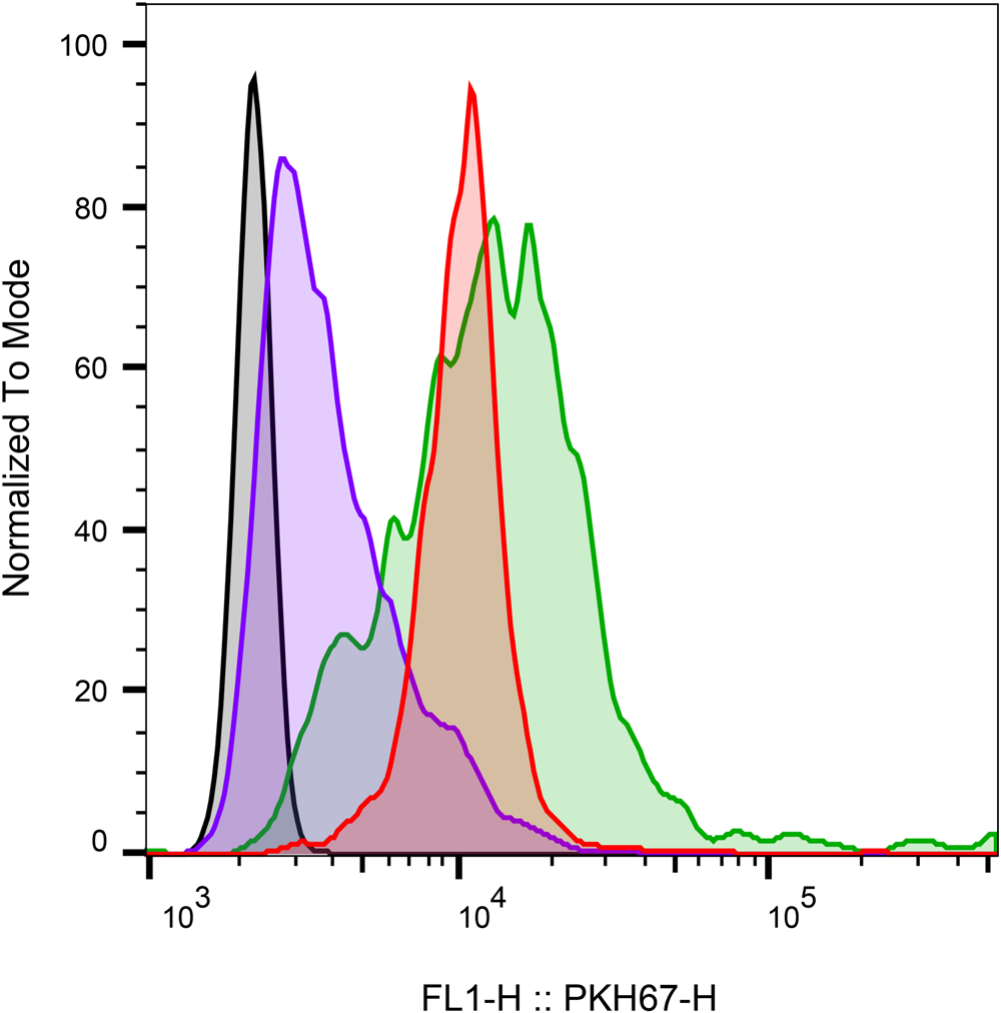
Representative pulse height histogram overlay of the fluorescent intensity (FI) of the membrane dye PKH67. Negative control and bEV samples were initially incubated with anti‐rgpB (against gingipain) conjugated beads and then further stained with PKH67. The figure shows the PKH67 median fluorescence intensity of rgpB conjugated beads incubated with the samples from ATCC 33277 (purple), A7A1‐28 (red), W83 (green), and negative control (black).

Flow cytometry was used to measure the fluorescence of PKH67 membrane‐stained gingipain‐captured bEVs to anti‐rgpB using magnetic beads (Figure 4). The delta median fluorescent intensity (ΔMFI) of bEVs captured from *P. gingivalis* ATCC 33277 was low (ΔMFI 1091) compared to the strains A7A1‐28 (ΔMFI 7917) and W83 (ΔMFI 6264) (Table 3).

**Table 3:** The delta median fluorescence intensity (ΔMFI) of PKH67 was measured with beads conjugated with anti‐rgpB (against gingipain) and incubated with bEV samples from (A) ATCC 33277, (B) A7A1‐28, (C) W83, and (D) negative control.

Label	Bacterial extracellular vesicles (bEVs) derived from	MFI	*ΔMFI*
A	ATCC 33277	3407	1091
B	A7A1‐28	10,233	7917
C	W83	8580	6264
D	Negative control	2316	

**Figure 5:**
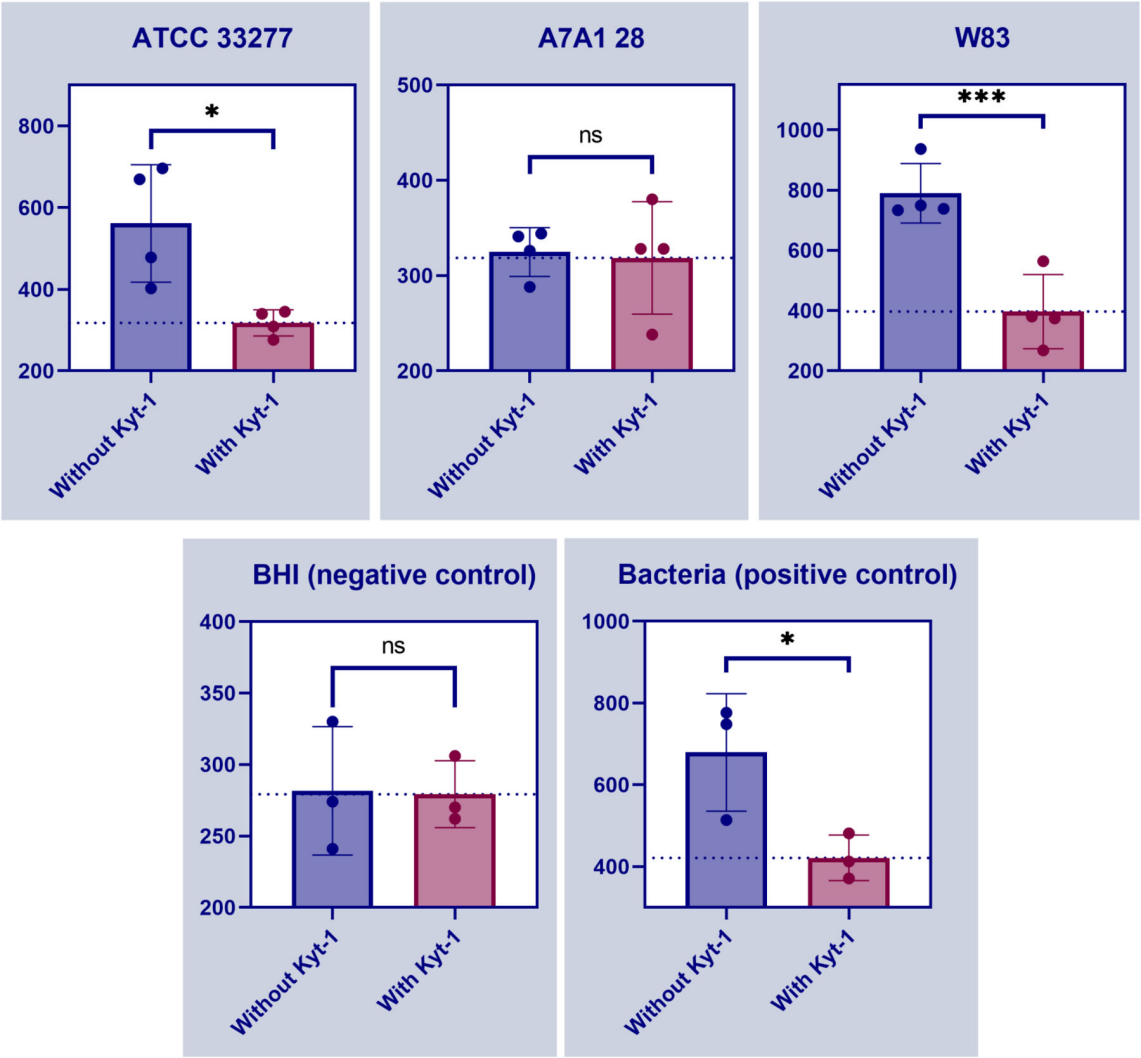
Gingipain activity and its inhibition with KYT‐1 of bacterial extracellular vesicles (bEVs) isolated from ATCC 33277, A7A1‐28, W83, and negative control and positive control (bacterial strain 195PG3) (*p < 0.05; ***p < 0.001).﻿﻿

Assessing the enzymatic activity of gingipain in bEVs compared to controls using fluorescence enzyme assay showed activity in bEVs from two of the bacterial strains (ATCC 33277 and W83) compared to the negative control. This was compatible with enzyme activity, as these two strains showed cleavage activity when compared to negative control indicating gingipain activity (Figure 5). Adding the inhibitor KYT‐1 showed inhibited gingipain activity both for strain ATCC 33277 and W83 and also for the positive control, validating the enzyme activity as expected (Figure 5). There was no observed enzyme activity for A7A1‐28 or the negative control. Experiments performed on the whole bacteria (OD600) show the enzymatic activity of gingipains for all three strains, including A7A1‐18 (not shown). Samples from ATCC 33277 showed relative fluorescence per minute of 1350 ± 45.0, A7A1‐28 1591 ± 16.0, and W83 1641 ± 28.0. In comparison, the negative control showed a relative fluorescence of 442 ± 11, which gives over a three‐fold increase for all three strains of whole bacteria, indicating gingipain activity.

### 3.3 Uptake of bEVs in oral fibroblasts

**Figure 6:**
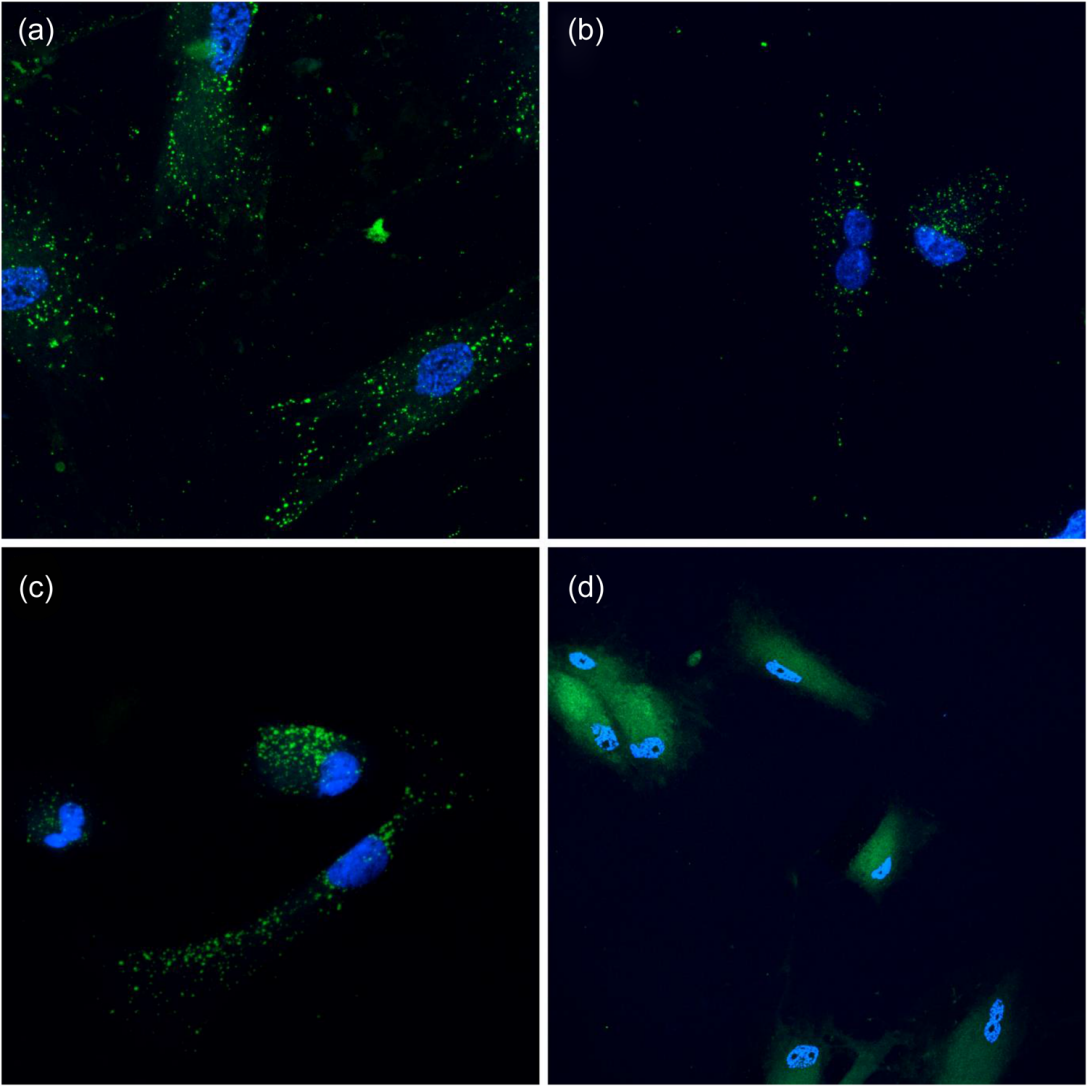
Visualization of bacterial extracellular vesicles (bEVs) and negative control in oral fibroblasts with confocal microscopy. Representative confocal pictures of uptake of bEVs from (a) ATCC 33277, (b) A7A1‐28, (c) W83, and (d) negative control. Fluorescent dye (green; PKH67) indicates intracellular localization in the cytoplasm consistent with bEV internalization when comparing the intensity and distribution of green color in the cells exposed to bEVs compared to negative control.

Confocal microscopy of oral fibroblasts stained with DAPI and incubated with PKH67 labeled bEVs from *P. gingivalis* displayed cytosolic labeling consistent with bEV internalization (Figure 6). We observed the nucleus (blue) and the typical elongation of the fibroblasts. Vesicle‐like structures with high fluorescence intensity were visible in the fibroblasts exposed to bEVs (Figure 6a–c) compared to fibroblasts exposed to negative control samples (Figure 6d), which displayed less intense fluorescence. There was no visible or consistent difference in the number of particles taken up by the fibroblasts, neither over time (2, 4, and 6 h) nor between the three strains, compared to the negative control.

## 4 DISCUSSION

In this work, we optimized and adapted our existing techniques for EV isolation to characterize bEVs from *P. gingivalis*. In addition, we confirmed the presence of the virulence factor RgpB in bEVs and explored the uptake of bEVs into oral fibroblasts. The isolated vesicles will in the future be used to scrutinize the effect of *P. gingivalis* bEVs in oral tissue.

### 4.1 Choice of strains

Various studies characterize the virulence of different *P. gingivalis* strains. The major fimbrial gene *fimA*, which contains six main genotypes, has been used to screen populations of periodontal disease. From those studies, the indication of high versus low virulence appears, as *fimA* type Ib, II, and IV are more commonly associated with periodontal disease, whereas types I and III are found primarily in isolates from healthy individuals (Enersen et al., 2008; Zhao et al., 2007). In a mouse model, *fimA* type II and IV were cytotoxic and invasive, while strains with *fimA* type I and III were less inflammatory and more likely to form localized self‐limiting infections (Nakano et al., 2004).

*P. gingivalis* strains interact differently with fibroblasts due to fimbriae variations, the presence or absence of capsules, and different kinetics of arg‐gingipain (Seers et al., 2020). ATCC 33277, with *fimA* I genotype, noncapsulated represents a strain with fairly low virulence (but not avirulent), that induces a primary immune response like localized periodontal abscesses (Laine & Winkelhoff, 1998). W83 expresses a very low level of *fimA* fimbriae (genotype IV) but is encapsulated (K1), causing spreading infection (Laine & Winkelhoff, 1998) and giving a persistent immune response, thus representing high virulent strains, as is also true for A7A1‐28 with *fimA* (genotype II) and capsule (K3). In macrophages, W83 and ATCC 33277 invade, but only W83 can escape and re‐enter cells (Werheim et al., 2020).

Since the majority of clinical *P. gingivalis* isolates are encapsulated (Laine et al., 1997) vesicles were harvested from *P. gingivalis* from two different capsular serotypes (W83 and AA7A1‐28) in addition to the noncapsulated ATCC 33277 strain (Laine et al., 1997).

### 4.2 Isolation and characterization

In the present study, we demonstrate the successful isolation and characterization of bEVs by applying a robust combination of methods.

The bEVs were isolated in the late exponential phase of bacterial growth, as it is known that the bacteria secrete a higher amount of vesicles in this phase (Naradasu et al., 2021). Still, our experience was that the number of vesicles differed also when isolated at the same OD value, even though we cultured and isolated the vesicles in the same manner every time. Several factors are known to affect the secretion and content of bEVs, such as nutrient availability, pH, and temperature (Lynch et al., 2019; McBroom & Kuehn, 2007; McMahon et al., 2012; Schulz et al., 2020; Toyofuku et al., 2014). These factors were to the best of our ability kept constant in our experiments. Thus, the reason for the variation in vesicle numbers in our study could be due to natural variations of bacterial vesicle blebbing (Kulp & Kuehn, 2010; Naradasu et al., 2021; Xie, 2015).

There are several methods available for isolating bEVs from bacteria, and the choice of method is considered a key step in EV studies (Coumans et al., 2017). No method provides complete separation of pure EVs, and one technique alone is not enough to fully characterize the vesicles (Witwer et al., 2021). Thus, isolated bEVs from *P. gingivalis* grown in BHI broth were characterized with NTA, TEM, and flow cytometry. Different isolation methods give different outcomes in both purity and yield (Veerman et al., 2021). Combining several techniques when isolating EVs is recommended, as we did by combining SEC and ultrafiltration (Nordin et al., 2015; Onódi et al., 2018). This is a preferred combination, confirmed by several studies to give a predictable outcome (Nordin et al., 2015; Onódi et al., 2018). In addition, the methods are relatively easy to use. Stam et al. published an overview of isolation techniques showing that 60% of the published papers in 2019 were based on combining two or more isolation methods and concluded that a combination of isolation techniques gives more pure samples (Stam et al., 2021). Although small lipoproteins, a known contaminator in SEC fractions, might have been present in our study, the removal of lipoproteins is shown to be more efficient when combining more than one isolation technique (Karimi et al., 2018).

The BHI broth used as a bacterial growth broth in this study contains components from beef heart and calf brains and may include vesicles and vesicle‐like structures (Le et al., 2023). We did not find any vesicle‐like particles in the negative control samples with TEM, but we cannot conclude that the broth is vesicle‐free. On the other hand, since we consistently found gingipains in the bacterial vesicles, we can confirm that these vesicles are derived from the bacteria and not from the broth.

For both the bEV samples and the negative controls, NTA showed that nanoparticles were present in similar quantities. However, no visible vesicle‐like particles were found for the negative control alone after SEC and TEM. The size tended to be smaller in negative control samples than in the bEV samples containing BHI broth. The particles of the BHI broth are also expected to be present in the samples with bEVs but may together with other undefined particles be masked by the larger bEVs due to the inherent nature of NTA analysis. The detected components in the negative control, that is BHI broth without bacteria, may represent nutrients for bacterial growth. When bacteria are not present, these nutrients will be unused and thus visible at NTA, as shown. Therefore, it is not possible to completely distinguish the different particle populations from each other (Giebel & Helmbrecht, 2017; Guerreiro et al., 2018). Le et al. (2023) detected dark structures on TEM pictures of EV‐like particles (EVLPs) from BHI, but after using eukaryotic EV markers they concluded that the EVLPs in BHI probably are large protein aggregates and not true EVs. Finally, several other factors can affect the NTA results, among them the potential fusion of vesicles (Grava et al., 2022). Different selections of threshold and camera levels during NTA analysis will also affect the outcome (Shao et al., 2018). We based our settings on previous experiments (Guerreiro et al., 2018) and kept them unchanged for all our analyses. Since it is not fully understood if or how both these vesicle‐like particles and lipoproteins already present in culture broth will influence functional studies using EVs, one should keep this point in mind and be sure to include controls. Likewise, this should be remembered when comparing results from different papers.

An additional methodological point to consider is that the sample preparation before TEM imaging may change the visible structure of EVs (Cizmar & Yuana, 2017). It is known that TEM pictures of EVs may vary, even though they are taken from the same samples and with the same magnification. Nonetheless, we repeatedly observed particles with size, shape, and structure consistent with EVs, although the TEM pictures were heterogeneous. Zaboe et al. discuss the diverse morphology of EVs from the same cell and suggest that it may be due to the presence of different vesicle subpopulations (Zabeo et al., 2017). Lobb et al. mention that cup‐shaped EVs can be observed, and suggest that it may be due to the dehydration of samples (Lobb et al., 2015). An additional methodological point to consider is that the sample preparation before TEM imaging may change the visible structure of EVs (Cizmar & Yuana, 2017).

Finally, in the present study, we consistently used vesicles stored in PBS at −80°C, as recommended in the literature (Jeyaram & Jay, 2017). However, no consensus exists for optimal storage conditions (Witwer et al., 2013) and there is no consensus either as to what degree freezing and thawing of bEVs can alter the outcome of experiments. We did not experience that freeze‐thaw cycles influenced our flow cytometry and arg‐gingipain assay results, although we did not systematically compare the results with fresh vesicles. When we used fresh bEVs as an additional control, there were no signs of a lack of activity due to the freeze‐thaw. A few cycles of freeze‐thaw were used for practical reasons, although the advice for functional studies is to use fresh rather than frozen vesicles (Lőrincz et al., 2014).

### 4.3 Verification of gingipains and gingipain activity in bEVs

Identifying a virulence factor specific to *P. gingivalis* is of great advantage when verifying the bEVs. As described in the results, flow cytometry and a gingipain assay both demonstrated the presence of gingipains and thus the bacterial origin of the vesicles. In flow cytometry, the magnetic beads with the bEVs attached were mildly sonicated. Sonification of samples before flow cytometry analysis is common (Nizamudeen et al., 2021) but it is not known to what degree this could affect the vesicles and thus the result, and in what amount possible aggregates are disassembled. Sonication of our samples increased the number of events, thus more particles were analyzed, indicating that aggregates existed in the samples before sonication. To explore the enzymatic activity of the gingipains present in the bEVs, a fluorescence‐based activity assay with a specific substrate for arg‐gingipain was used. The relative fluorescence indicated enzyme activity. Consistent with several other studies (Mantri et al., 2015; Nara et al., 2021; Veith et al., 2018), we found bEVs from *P. gingivalis* to contain gingipain by flow cytometry, and through the enzyme assay, we confirmed that the gingipains were active in two of the three strains (W83 and ATCC 33277). In the third strain (A7A1‐28), we confirmed the presence of gingipains, although enzymatic activity was not detectable. This was an unexpected finding since the whole bacteria showed enzymatic activity, and additional studies are needed to explore this missing activity. Of interest, Seers et al. (2020) point out that different strains of *P. gingivalis* whole bacteria show different gingipain activity in a kinetic study, and that A7A1‐28 shows significantly lower activity compared to ATCC 33277 and W50, a strain similar to W83. Castillo et al. (2022) showed that bEVs from ATCC 33277 demonstrated higher Rgp‐activity compared to W83. Furthermore, gingipain maturation/activation can be modified by acetylation (Dou et al., 2015; Mishra et al., 2018) and regulation of their C‐terminal domain (Veith et al., 2020). Gingipains themselves can also have different transpeptidase as well as cysteine protease activity (Seers et al., 2020). Thus, it is possible that such modifications could have influenced our result. To the best of our knowledge, there are no other published studies on gingipain activity in bEVs from A7A1‐28.

### 4.4 Uptake of bEVs into oral fibroblasts

Understanding the uptake of bEVs from perio‐pathogenic bacteria is still in its infancy. We aimed to determine the uptake of bEVs into oral fibroblasts. In the early stages of our study, we explored how different concentrations of bEVs and various times of incubation influenced the uptake. We chose the concentration of bEVs that gave the most consistent results. While performing the experiment, it became evident that the fibroblasts at times displayed atypical morphology after vesicle uptake at high vesicle concentration, indicating that the fibroblasts possibly responded negatively to vesicle exposure. Bartruff et al. reported a dose‐dependent inhibition of growth and proliferation of human gingival fibroblasts after exposure to bEVs from *P. gingivalis* and suggested that bEVs contributed to chronic periodontitis by suppressing fibroblast proliferation and fibroblast growth, as the presence of fibroblasts is important for maintaining and repairing gingival tissue (Bartruff et al., 2005). The confocal images of our study indicated that the vesicles were internalized, although it could not be ruled out completely whether they were merely attached to the membrane (Arasu et al., 2019). Earlier studies have shown the ability of bEVs from *P. gingivalis* to enter cells such as gingival fibroblast (Mantri et al., 2015; O'Donoghue & Krachler, 2016). The cellular effect of vesicle uptake and the pathway of entry are not fully understood. EV uptake can involve several pathways and be as heterogeneous as the origin of vesicles. Some studies have focused on how the labeling of vesicles with lipophilic dyes, such as the widely used PKH dye, can give false positive results through concurrent labeling of remaining proteins and lipoproteins in addition to the bEVs (Karimi et al., 2018; Simonsen, 2019). Studies have also illustrated how PKH can form aggregates that can be taken up by cells and, thus, be problematic to differentiate from EVs (Dehghani et al., 2020; Simonsen, 2019). This emphasizes the importance of proper controls for comparison, as our use of BHI broth as negative control and using a specific antibody against gingipain both in vesicle and broth control samples.

In this study, we isolated and characterized bEVs from three strains of *P. gingivalis*. We confirmed the presence of vesicle gingipains in all three strains and the activity of gingipains in two of three strains, and demonstrated the uptake of bEVs into oral fibroblast. This suggests a possible influence of bEVs from *P. gingivalis* on oral fibroblasts. Future studies are needed to understand how bEVs affect cells such as oral fibroblasts, and how this could influence the pathogenesis of periodontitis compared to uptake of the bacteria alone. Further investigations should also focus on the activity of gingipains and the effects of vesicle‐bound gingipains compared to bacterial‐bound gingipains during the early and late exponential phases.

## AUTHOR CONTRIBUTIONS

**Helene R. Haugsten**: Conceptualization (equal); data curation (lead); formal analysis (lead); investigation (lead); methodology (equal); software (lead); validation (lead); visualization (lead); writing—original draft (equal). **Anne K. Kristoffersen**: Conceptualization (equal); formal analysis (equal); investigation (equal); methodology (equal); project administration (equal); resources (equal); supervision (equal); validation (equal); visualization (equal); writing—original draft (equal). **Trude Haug**: Conceptualization (equal); formal analysis (equal); funding acquisition‐supporting, investigation (equal); methodology (equal); project administration (equal); resources (equal); supervision (equal); validation (equal); visualization (equal); writing—original draft (equal). **Tine M. Søland** Conceptualization (equal); formal analysis (equal); funding acquisition‐supporting, investigation (equal); methodology (equal); project administration (equal); resources (equal); supervision (equal); validation (equal); visualization (equal); writing—original draft (equal). **Reidun Øvstebø**: Formal analysis (supporting); methodology (equal); software‐supporting; writing—original draft (supporting). **Hans C. D. Aass**: Data curation (equal); formal analysis (equal); investigation (equal); methodology (equal); software (equal); validation (equal); writing—original draft (equal). **Morten Enersen**: Conceptualization (equal); formal analysis (equal); funding acquisition‐supporting, investigation (equal); methodology (equal); project administration (equal); resources (equal); supervision (equal); validation (equal); visualization (equal); writing—original draft (equal). **Hilde Galtung**: Conceptualization (equal); formal analysis (equal); funding acquisition (lead); investigation (equal); methodology (equal); project administration (lead); resources (equal); supervision (lead); validation (equal); visualization (equal); writing—original draft (equal).

## CONFLICT OF INTEREST STATEMENT

None declared.

## ETHICS STATEMENT

None required.

## Data Availability

Data are available in the Zenodo repository at https://doi.org/10.5281/zenodo.8402964.
